# Interleukin-22 Mediates the Chemotactic Migration of Breast Cancer Cells and Macrophage Infiltration of the Bone Microenvironment by Potentiating S1P/SIPR Signaling

**DOI:** 10.3390/cells9010131

**Published:** 2020-01-06

**Authors:** Eun-Young Kim, Bongkun Choi, Ji-Eun Kim, Si-On Park, Sang-Min Kim, Eun-Ju Chang

**Affiliations:** 1 Department of Biomedical Sciences, Asan Medical Center, University of Ulsan College of Medicine, Seoul 05505, Korea; kimberly_kim44@hotmail.com (E.-Y.K.); bkchoi89@hanmail.net (B.C.); kge33@sookmyung.ac.kr (J.-E.K.); sionirena@naver.com (S.-O.P.); rlatkals90@gmail.com (S.-M.K.); 2 Stem Cell Immunomodulation Research Center, Asan Medical Center, University of Ulsan College of Medicine, Seoul 05505, Korea; 3 Department of Biochemistry and Molecular Biology, Asan Medical Center, University of Ulsan College of Medicine, Seoul 05505, Korea

**Keywords:** interleukin 22, breast cancer, IL22R1, S1P, S1PR1, metastasis

## Abstract

The interleukin-22 (IL-22) signaling pathway is well known to be involved in the progression of various cancer types but its role in bone metastatic breast cancer remains unclear. We demonstrate using human GEO profiling that bone metastatic breast cancer displays elevated interleukin-22 receptor 1 (IL-22R1) and sphingosine-1-phosphate receptor 1 (S1PR1) expression. Importantly, IL-22 stimuli promoted the expression of IL-22R1 and S1PR1 in aggressive MDA-MB-231 breast cancer cells. IL-22 treatment also increased sphingosine-1-phosphate production in mesenchymal stem cells (MSCs) and induced the sphingosine-1-phosphate (S1P)-mediated chemotactic migration of MDA-MB-231 cells. This effect was inhibited by an S1P antagonist. In addition to the S1PR1 axis, IL-22 stimulated the expression of matrix metalloproteinase-9 (MMP-9), thereby promoting breast cancer cell invasion. Moreover, IL-22 induced IL22R1 and S1PR1 expression in macrophages, myeloid cell, and MCP1 expression in MSCs to facilitate macrophage infiltration. Immunohistochemistry indicated that IL-22R1 and S1PR1 are overexpressed in invasive malignant breast cancers and that this correlates with the MMP-9 levels. Collectively, our present results indicate a potential role of IL-22 in driving the metastasis of breast cancers into the bone microenvironment through the IL22R1-S1PR1 axis.

## 1. Introduction

Interactions between tumor cells and the host cells of the surrounding tumor microenvironment play an important role in tumor development, including cancer initiation, progression, and invasiveness [1]. The mesenchymal stem cells (MSCs) and macrophages are a particularly important component of the tumor microenvironment [2,3]. It has been suggested that MSCs promote tumor development and metastasis through several processes including the release of growth factors, enhancement of invasiveness and metastasis via the production of matrix metalloproteinases, and enabling immune evasion by providing an immunosuppressive environment [4,5,6]. Macrophages are also multi-functional in the breast tumor microenvironment where they can stimulate breast tumor progression, including cancer initiation, angiogenesis, invasion, and metastasis. These cells are required for the early dissemination of cancers and can promote a metastatic cascade by generating an immunosuppressive tumor microenvironment via the production of cytokines, chemokines, and growth factors [3,7]. Although recent reports have emphasized the importance of the tumor microenvironment in host organs and its activation by signals from disseminated cancer cells, which is designated as a “pre-metastatic niche” [8], the details of the molecular mechanisms by which breast cancer cells are programmed into metastasis seeds and interact with bone microenvironments in association with MSCs and macrophages remain to be elucidated. 

Interleukin-22 (IL-22) is a key effector cytokine that plays a role in the pathogenesis of several inflammatory diseases including cancer [9]. Several lines of evidence have suggested that IL-22 signaling may regulate tumor progression by promoting cell growth and survival [10,11,12]. In further support of this, the aberrant expression of the IL-22 cognate receptor, interleukin-22 receptor 1 (IL-22R1), has been observed in many types of tumor, including lymphomas [13] and pancreatic [14], lung [15], and colon cancers [16]. Of note, the ablation of the IL-22 pathway via an endogenous antagonist, IL-22-binding protein, suppressed tumorigenesis in a genetic model [17] and another study has reported that blocking IL-22 which was locally produced by innate lymphoid cells inhibits colorectal tumors in the mouse [18]. Moreover, another prior study has indicated that IL-22 was upregulated in human Edmondson Grade III-IV hepatocellular carcinoma (HCC) and that IL-22R1 was highly expressed in aggressive HCC cases [19]. Other data have revealed that increased IL-22 is related to the growth and metastasis of gastric cancer [20]. However, despite these various observations implicating the IL-22/IL22R signaling system as a potential marker for aggressive cancer, the precise mechanism by which such an axis promotes the motility of cancer cells and their metastasis at different organ sites has not been definitively identified, especially in metastatic breast cancer.

Sphingosine kinase 1 (SphK1), sphingosine-1-phosphate (S1P), and S1P receptors (S1PRs) expression have been suggested to be associated with progression to advanced tumor stages in estrogen (ER)-positive breast cancers in vivo [21]. S1P is a bioactive lipid mediator that mediates various cellular functions, including proliferation, migration, survival, and angiogenesis. S1P is produced by SphK1 and interacts with components in the tumor microenvironment, which may regulate breast cancer metastasis [22]. SphK1 also contributes to the growth, metastasis, and chemoresistance of various human cancers [23,24]. Binding of S1P to cell surface S1PRs activates signaling in cytoplasm and gene activation in nucleus in autocrine and paracrine manners [21,25]. There are five S1P cell surface G-protein coupled receptor subtypes, S1PR1~5; each of these couples with various G proteins and specific downstream targets [26,27]. For example, the S1PR1 couples through Gi/o; S1PR2/S1PR3 through Gi/o, Gq, and G12/13; and S1PR4/S1PR5 through Gi/o and G12/13 [27]. In ER-positive breast cancer patients, high membrane S1PR1 expression was associated with shorter time to recurrence, whilst high cytoplasmic S1PR1 was correlated with shorter disease-specific survival times [28], suggesting a cancer promoting role of S1PR1 subtype.

In our present study, we analyzed the potential role of the IL-22 axis in the association between aggressive MDA-MB-231 breast cancer cells and the bone environment, particularly in relation to MSCs and macrophages. Intriguingly, we found from our analyses that the IL-22-IL-22R1 cascade has a potential role in mediating the invasion of breast cancer cells via elevated sphingosine-1-phosphate receptor 1 (S1P1R)-matrix metalloproteinase-9 (MMP-9) activity. Furthermore, we observed that IL-22 signaling increased sphingosine-1-phosphate (S1P) production in MSCs and induced the S1P-mediated chemotactic motility of MDA-MB-231 cells towards MSCs. In addition, this invasive motility of metastatic cancer cells was dramatically suppressed by S1PR1-specific inhibition. Notably, IL-22 stimulation enhanced the expression of monocyte chemoattractant protein 1 (MCP-1) in MSCs, which contributes to the macrophage accumulation required for metastatic cancer progression. Taken together, our current results suggest that the induction of the IL-22R1-S1PR1 axis is sufficient to drive the metastasis of aggressive breast cancers into bone. 

## 2. Materials and Methods

### 2.1. Reagents and Cell Cultures

Recombinant human protein, IL-22 and the CCL2/MCP-1 antibody for human chemokine (C-C motif) ligand 2, which is also referred to as monocyte chemoattractant protein 1 (MCP-1), were purchased from R&D systems (Minneapolis, MN, USA). Human macrophage colony-stimulating factor was obtained from PeProtech (Rocky Hill, NJ, USA). The polyclonal anti-S1P1/EDG1 antibody and MMP-9 inhibitor were sourced from Abcam (Cambridge, UK). VPC24192 (S1P agonist) and VPC23019 (S1P antagonist) were obtained from Avanti polar lipids (Alabaster, AL). Fetal bovine serum (FBS) and non-essential amino acids were obtained from Life Technologies (Gaithersburg, MD, USA). All other chemicals were purchased from standard sources and were of molecular biology grade or higher. MCF-7, T47D, MDA-MB453, SKBR3, HCC1954, MDA-MB231, and MDA-MB157 cells were purchased from the American Type Culture Collection (Rockville, MD, USA). MDA-MB231-MT cells (highly bone-metastatic breast cancer cells, MDA-MB231 subline) were kindly provided by Dr Hong-Hee Kim (Seoul National University, Seoul, Korea). Cells were maintained in DMEM or RPMI (GIBCO, Grand Island, NY, USA) supplemented with 10% FBS and antibiotic–antimycotic solution (Life Technologies) at 37 °C in a humidified atmosphere containing 5% CO_2_. Normal human bone marrow-derived mesenchymal stem cells (hBM-MSCs) were purchased from Lonza (Basel, Switzerland) and cultured in accordance with the manufacturer’s instructions using MSCGM^TM^ Bullet Kit (Lonza) complete growth media. Passage 3 to 4 hMSCs were used for all experiments.

### 2.2. RNA Isolation, RT-PCR, and qPCR

Total RNA was extracted from cultured hMSCs and various breast cancer cells using Trizol reagent (Invitrogen Life Technologies) following the manufacturer’s instructions. RevertAid First strand cDNA Synthesis kit (Thermo Fisher Scientific, Waltham, MA, USA) was used to synthesize cDNA from RNA and subsequent PCR was performed in a T100^TM^-Thermal Cycler (Bio-Rad, Hercules, CA, USA). The resulting PCR products were analyzed by electrophoresis in a 2% agarose gel and imaged using an ultraviolet gel imaging system (Bio-Rad). qPCR analysis was performed in optical 96-well plates using SYBR Green PCR master mix (Roche, Penzberg, Germany) and the Light Cycler 480 Real-time PCR Detection System (Roche), in accordance with the manufacturer’s instructions. Gene expression was normalized to that of glyceraldehyde-3-phosphate dehydrogenase (GAPDH), which was used as an internal control. The relative expression of the target genes was calculated by the standard curve method using the target Ct values and the Ct value for GAPDH.

### 2.3. Immunoblotting

Conditioned media (CM) from MDA-MB 231 or cell lysates were resolved by sodium dodecyl sulfate–polyacrylamide gel electrophoresis (SDS-PAGE) and electophoretically transferred to a polyvinylidene difluoride membrane (Bio-Rad). Non-specific interactions were blocked using 5% bovine serum albumin solution in Tris–buffered saline (20 mM Tris/HCl, pH 7.6, 150 mM NaCl, and 0.1% Triton X-100) for 1 h. The membranes were then incubated with the anti-S1PR1 or MMP-9 antibody overnight at 4 °C. Membranes were then incubated with the appropriate secondary antibodies conjugated with HRP and immunoreactivity was detected using an enhanced chemiluminescence detection kit (Millipore, Billerica, MA, USA).

### 2.4. ELISA

The secreted levels of S1P and MCP-1 protein in the CM of hMSCs were evaluated with a S1P- (MyBiosource, San Diego, CA), and MCP-1- (R&D Systems) specific sandwich ELISA system, following the manufacturer’s protocol.

### 2.5. Transwell Assay

Cell chemotaxis was assessed as described previously [29]. MDA-MB 231 cells (6 × 10^4^), either non-primed or primed by IL-22 (20 ng/mL), or bone marrow-derived macrophages (BMMs) (4 × 10^4^), were plated in the upper chamber of a 8- or 5 μm pore size Transwell system (Costar, Corning, NY, USA), respectively. To evaluate the chemotactic potential of S1P, the lower chamber received S1P agonist (200 Nm) or S1P antagonist (200 nM). To examine the chemotactic effect on MSCs of MCP-1 via the IL-22 signal axis, MSCs in the bottom chamber were pretreated with IL-22 for 24 h and further incubated with/without MCP-1 antibody (2 μg/mL), whilst the macrophages were loaded into the upper chamber. After incubation for 24 h to allow for macrophage migration, macrophages that had not migrated were scraped from the top of the membrane with a cotton swab, and the macrophages present in the lower chamber were stained with hematoxylin and counted under a microscope.

### 2.6. Matrigel Invasion Assay

To monitor the invasion potential of MDA-MB231 cells on an IL22-S1P-S1PR-MMP-9-lined axis, BD BioCoat Matrigel Invasion Chambers (BD Biosciences, San Jose, CA, USA) were used in accordance with the manufacturer’s instructions. Cells (5 × 10^4^) were primed with/without IL-22 for 24 h before seeding into the matrigel inserts. MSCs (2 × 10^5^) were also primed with IL-22 for 24 h and added to the lower chamber in medium with/without S1PR antagonist or MMP-9 inhibitor (MMP-9I). The plates were then incubated at 37 °C for 24 h. The filters were fixed in methanol and stained with hematoxylin and eosin. After removal of the contents of the upper membrane surface, invasive cells were counted microscopically in 10 random high-power fields per filter; each sample was assayed in duplicate and five independent assays were performed. In the inhibition experiments, cells were pretreated with inhibitors for 30 min and then evaluated in the invasion assay as detailed above. Representative phase-contrast pictures were obtained using an Olympus CKX53 microscope. Samples were analyzed in duplicate.

### 2.7. Immunohistochemical Staining Analysis

Human breast tumor tissue samples and corresponding normal tissues were purchased from US Biomax (Rockville, MD, USA). For antigen retrieval, formalin-fixed, paraffin-embedded sections were incubated in proteinase K (Biogenex, San Ramon, CA, USA) for 10 min prior to the application of a rabbit polyclonal anti-IL-22R/S1PR1/MMP-9 antibody (1:200 dilution; MBL Inc., Des Plaines, IL, USA; Cat. No. LS-A2102). After incubation with the primary antibody and a biotinylated secondary antibody, streptavidin-coupled alkaline phosphatase was applied. 3,3’-diaminobenzidine (DAB; Dako) was used as the chromogen and the sections were counterstained with hematoxylin.

### 2.8. Immunofluorescent Staining for S1PR1

Cells were grown on coverslips in 24-well plates for 24 h, washed twice with ice-cold phosphate-buffered saline (PBS) and fixed with 3% paraformaldehyde. After being blocked with 1% BSA in PBS for 30 min, the cells were incubated with a primary antibody against S1PR1 (1:100 dilution; Calbiochem) for 24 h at 4 °C. The cells were then washed three times with PBS and incubated with FITC-conjugated secondary antibody (1:200 dilution; Molecular Probes, Eugene, OR, USA). Nuclei were stained with 1 μg/mL DAPI. All images were obtained using a confocal laser scanning microscope (Carl Zeiss, Thornwood, NY, USA).

### 2.9. Statistics

The study results are presented as a mean  ±  SEM or mean ± SD. All quantitative experiments were performed at least three times independently. ANOVA, Student’s t test, post hoc, or Kruskal–Wallis tests were performed using GraphPad Prism 6 (La Jolla, CA, USA). ANOVA and Kruskal–Wallis tests were performed for two group comparisons and Tukey post hoc was used for multiple comparisons. Comparisons between Kaplan–Meier curves were performed using the log-rank test for survival analyses. * *p* < 0.05 was considered to indicate statistical significance.

## 3. Results

### 3.1. The Elevated Co-Expression of IL-22R1 and S1PR1 Is Associated with Advanced Human Breast Cancers with Bone Metastatic Potential

**Figure 1:**
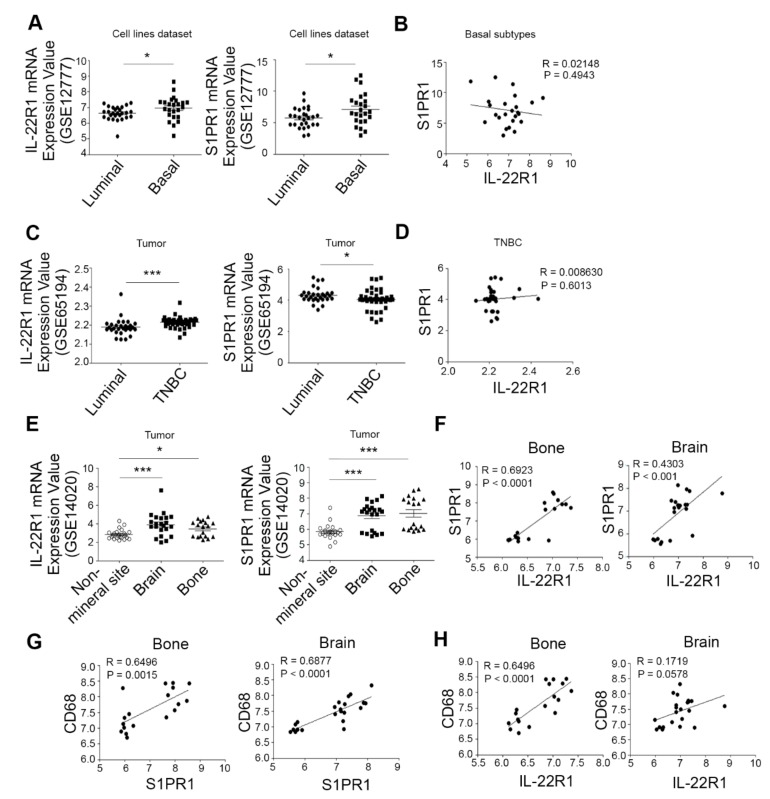
Breast cancers showing a correlation between interleukin-22 receptor 1 (IL-22R1) and sphingosine-1-phosphate receptor 1 (S1PR1) have a greater propensity to metastasize to bone. (**A**–**D**) IL-22R1 and S1PR1 mRNA levels were compared between the luminal and basal-like/triple-negative subtypes of human breast cancers using the chi-square test. Data were obtained from the GSE12777 and GSE65194 datasets of breast cancer cell lines (**A**) or from breast tumors (**C**). * *p* < 0.05 vs. luminal subtype. (**B**,**D**) Pearson’s correlation coefficient and linear regression array analysis of the correlation between IL-22R1 and S1PR1 expression in different human breast cancer subtypes. (**E**) IL-22R1 and S1PR1 expression in non-mineral site (lung and liver), brain, or bone metastasis-positive human breast cancer were compared using a chi-square test. The IL-22R1 (left) and S1PR1 (right) mRNA levels were obtained from the GSE14020 breast cancer dataset (*n* = 65). * *p* < 0.05, ** *p* < 0.005 vs. corresponding non-mineral organs. (**F**–**H**) Pearson’s correlation coefficient and linear regression array analysis of the correlation between IL-22R1 and S1PR1 (**F**), between CD68 and S1PR1 (**G**), and between CD68 and IL-22R1 (**H**) expression in bone and brain metastases from breast cancer. Values are expressed as a mean  ±  SD. Comparisons were performed using t-tests (two groups) or ANOVA (multiple groups).

To investigate the association between breast cancer development and the IL-22 receptor, IL-22R1 and S1PR1 expression signatures, we compared the mRNA expression of IL-22R1 and S1PR1 in luminal and basal/triple-negative subtypes of breast cancer cell lines and breast tumors. We utilized the published data from the Gene Expression Omnibus (GSE12777 and GSE65194) for this analysis. The IL-22R1 levels were significantly higher in the basal/triple-negative subtypes than in the luminal type (Figure 1A,C), indicating its elevated expression in more aggressive breast cancer. No correlation was observed however between the IL-22R1 and S1PR1 levels in the basal/triple-negative subtypes of breast cancer (Figure 1B,D). 

IL-22 has been suggested to regulate the progression of several tumors [10,11,12] but its involvement in breast cancer metastasis is largely unknown. To determine the potential involvement of elevated IL-22R1 and S1PR1 expression in breast cancer metastasis to distant organs, we analyzed a cohort of 65 breast cancer patients harboring a metastasis at a non-mineral site (lung and liver), brain, or bone. Gene expression data demonstrated that clinical breast cancer tissues from patients with a bone or brain metastatic status had higher IL-22R1 and S1PR1 levels compared to non-mineral metastatic breast cancer cases (*p* < 0.05, Figure 1E). In addition, there was a positive correlation between the expression of IL-22R1 and S1PR1 in bone or brain metastases in breast cancer patients (Figure 1F). However, the expression levels of IL-22, S1PR2, S1PR4, and S1PR5 showed no significant differences between lung, brain, bone, and liver metastases (Figure S1). In addition, the level of CD68 transcript expression which represents macrophage infiltration was higher in the basal/triple-negative subtypes than in the luminal type (Figure S1). Bone or brain metastatic status had higher CD68 level compared to non-mineral metastatic breast cancer cases (Figure S1). Moreover, we observed the positive correlation between the expression of S1PR1 and CD68 (Figure 1G) and between IL-22R1 and CD68 (Figure 1H) in bone or brain metastases in breast cancer patients. Triple-negative subtype of breast tumors expressed higher levels of MCP1 and CD14 than those of luminal subtype breast tumors and bone metastatic status exhibited higher MCP1 and CD14 levels compared to brain metastatic status (Figure S1). Collectively, these results suggest that IL-22R1 and S1PR1 are elevated in breast cancers with bone metastatic properties.

### 3.2. IL-22 Enhances IL-22R1 and S1PR1 Expression in Invasive Metastatic Breast Cancer Cells

**Figure 2:**
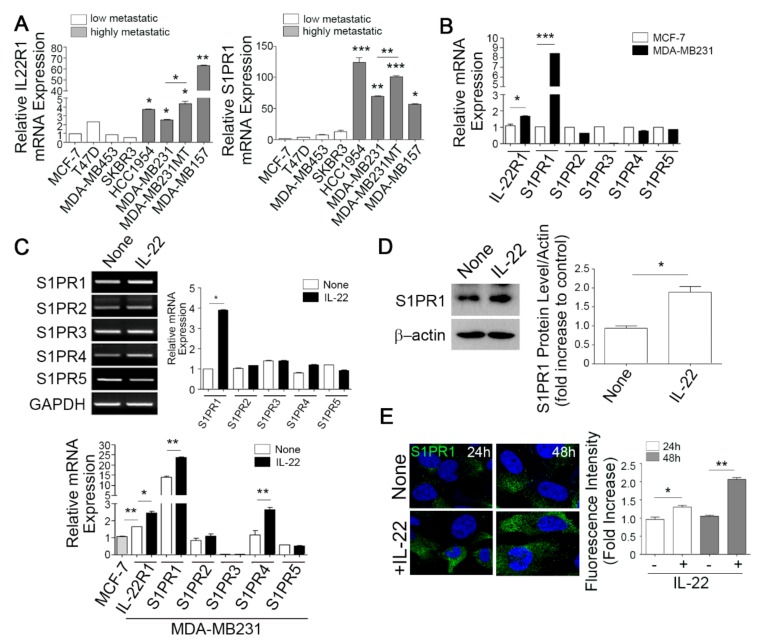
Interleukin-22 (IL-22) enhances IL-22R1 and S1PR1 expression in invasive metastatic breast cancer cells. (**A**) IL-22R1 (left) and S1PR1 (right) expression was analyzed by qPCR in low metastatic (MCF-7, T47D, MDA-MB453, and SKBR3) and highly metastatic (HCC1954, MDA-MB231, MDA-MB157, and MDA-MB231-MT) breast cancer cell lines. * *p* < 0.05, ** *p* < 0.005, and *** *p* < 0.001 vs. non-metastatic cancer cells, MCF-7. (**B**) Quantification of IL-22R1, S1PR1, S1PR2, S1PR3, S1PR4, and S1PR5 expression levels in MCF-7 and MDA-MB231 cells by qPCR. * *p* < 0.05, *** *p* < 0.001 vs. MCF-7 cells. (**C**) Both IL22R1 and S1PR1 expression is dependent on the IL-22 signaling axis. MDA-MB231 cells were stimulated with IL-22 for 24 h and S1PR1-5 transcript levels were analyzed by RT-PCR (top) or qPCR (bottom). Densitometric quantification of the S1PR1~5 transcripts is represented. * *p* < 0.05, ** *p* < 0.005 vs. MCF-7 cells. (**D**) MDA-MB231 cells were treated with IL-22 for 24 h and S1PR1 protein expression was determined by immunoblotting (left). Densitometry quantification of S1PR1 compared to β-actin was then performed (right). * *p* < 0.05 vs. non-treated control group. (**E**) Cells were stained using the antibody DyLight™ 488 Conjugated (for S1PR1; green) and nuclei were counterstained (DAPI, blue) and observed by confocal microscopy. Scale bar, 100 μm. Quantification of the S1PR1 fluorescence intensity is represented. Data are presented as the mean ± standard deviation from three independent experiments. Comparisons were performed using t-tests (two groups) or ANOVA (multiple groups).

Given our observations of the elevated co-expression of IL-22R1 and S1PR1 in aggressive human breast cancer, we further compared their expression between high and low metastatic breast cancer cell lines. IL22R1 and S1PR1 transcripts were detectable at only low levels in low metastatic breast cancer cell lines (MCF-7, T47D, MDA-MB453, and SKBR3) but showed robustly increased levels in the highly metastatic breast cancer cell lines (HCC1954, MDA-MB231, and MDA-MB157) as determined by qPCR (Figure 2A). Moreover, MDA-MB231MT, subclone of breast cancer cell lines that can form metastases in the bones expressed higher levels of IL-22R1 and S1PR1 than those of MDA-MB231 cell line (Figure 2A). In contrast, the expression levels of S1PR2-5 showed minimal differences between the low metastatic MCF-7 and high metastatic MDA-MB231 cells (Figure 2B). 

The positive correlation we observed between IL22R1 and S1PR1 (Figure 1 and Figure 2A) raised the possibility that IL-22 signaling may modulate S1PR1 expression. We thus explored the possibility of an IL-22 dependent induction of S1PR1 expression in MSCs. IL-22 treatment increased the IL-22R1 and S1PR1 mRNA levels, which was accompanied by an upregulation of S1PR4 in MDA-MB231 cells as demonstrated by RT-PCR and qPCR (Figure 2C). In comparison, IL-22 did not affect the expression of S1PR2, S1PR3, or S1PR5 (Figure 2C). IL-22R1 and S1PR1 transcript levels were also enhanced by IL-22 treatment in HCC1954 cells whilst IL-22 treatment failed to increase IL-22R1 and S1PR1 transcript levels in MCF-7 cells (Figure S2). Highly metastatic breast cancer cells expressed elevated protein levels of pERK and pSTAT3 compared to low metastatic breast cancer cells and IL-22R1 silencing led to reduced protein levels of pERK and pSTAT3 upon IL-22 exposure (Figure S3). In addition, IL-22-induced S1PR1 mRNA expression was significantly inhibited by PD98059 (ERK inhibitor) and STAT3i (STAT3 inhibitor) in MDA-MB231 cells, however, MCF-7 cells showed no significant difference (Figure S3). Consistent with this modulation of gene transcription, IL-22 treatment induced S1PR1 protein expression as determined by immunoblotting (Figure 2D) and immunofluorescent staining further verified the IL-22 mediated induction of S1PR1 protein expression in MDA-MB231 cells (Figure 2E). By contrast, the expression of sphingosine kinase 1 protein, which phosphorylates sphingosine to sphingosine-1-phosphate (S1P), was not affected by IL-22 in MDA-MB231 cells (data not shown). Taken together, these results indicate that IL-22 may serve as an important determinant of S1PR1 induction in breast cancer cells.

### 3.3. IL-22-Induced S1P in Mesenchymal Stem Cells (MSCs) Promotes the Migratory and Invasive Potential of Breast Cancer Cells

**Figure 3:**
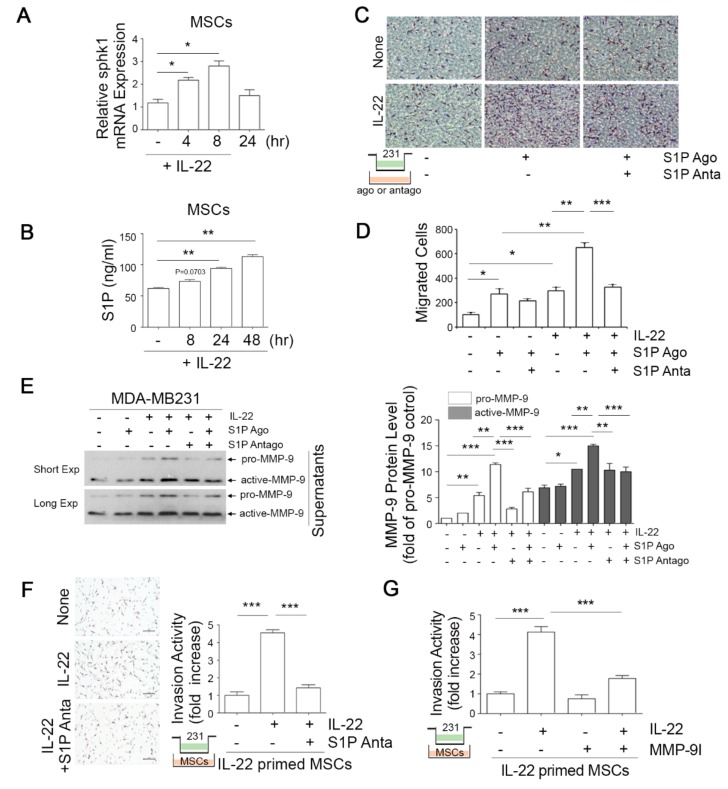
IL-22-induced sphingosine-1-phosphate (S1P) in mesenchymal stem cells (MSCs) promotes the migratory and invasive potential of breast cancer cells. (**A**,**B**) MSCs were treated with IL-22 for the indicated times and sphingosine 1 kinase mRNA expression was then analyzed by qPCR (**A**) and the S1P protein level in CM was measured by ELISA (**B**). * *p* < 0.05, ** *p* < 0.005 vs. control group. (**C**,**D**) Inhibition of the S1P axis suppresses the increase in migration and invasiveness evoked by IL-22 in MDA-MB231 cells. In the inhibition experiments, MDA-MB231 cells primed by IL-22 were loaded into the upper chamber of a Transwell system and incubated with or without 1 μM of S1PR agonist (Ago) or antagonist (Anta) in the lower chamber. After 24 h, the number of migrating cells was analyzed (**C**) and quantified (**D**). (**E**) IL-22 activates MMP-9 in MDA-MB231 cells. MDA-MB231 cells primed with IL-22 or untreated were incubated with or without S1P agonist or antagonist. The supernatants were harvested and MMP-9 protein expression was analyzed by immunoblotting. The densitometric quantification of the MMP-9 protein signal is represented. (**F**) MDA-MB 231 cells primed by IL-22 were loaded into the upper chamber of the Transwell system and MSCs were stimulated with IL-22 for 24 h in the lower chamber. In the inhibition experiments, these cells were co-cultured with or without 1 μM of S1P antagonist in the lower chamber. After 24 h, the number of cells that had migrated through the matrigel matrix was analyzed (left) and quantified (right). Representative invasive images from the co-culture system are shown. *** *p* < 0.001. (**G**) MMP-9 inhibitor prevents IL-22-induced breast cancer cell migration towards MSCs. IL-22-primed MDA-MB231 cells were loaded into the upper chamber of a co-culture Transwell system and incubated with or without 10 μM MMP-9 inhibitor (MMP-9I) in the lower chamber. The number of cells that had migrated to the lower chamber was determined. *** *p* < 0.001. The results represent the mean  ±  SEM of at least three independent experiments. Comparisons were performed using ANOVA.

Given our current observations that both IL-22R1 and S1P1R expression is elevated in bone metastatic breast cancer (Figure 1), we investigated whether IL-22 stimulates the S1P signaling pathway in human bone marrow-derived mesenchymal stem cells (hBM-MSCs), which reside in the bone environment. Unlike the situation in MDA-MB231 cells, IL-22 exposure markedly augmented the sphingosine kinase 1 mRNA level in MSCs (Figure 3A), which was accompanied by increased S1P protein expression (Figure 3B). IL-22 exerted no effect on the S1PR1-5 transcript levels in these cells however (data not shown). Although the expression of IL-22 was found to be elevated in aggressive breast cancer cells (Figure 2), the role of this cytokine in breast cancer metastasis remained unclear. We therefore examined whether IL-22 and/or S1P signaling would induce the migration of breast cancer cells using a Transwell assay. Exogenous IL-22 or S1PR1 agonist alone enhanced the migratory capacity of MDA-MB231 cells by approximately 3.5-fold compared with untreated cells (Figure 3C,D). Of note in this regard, the simultaneous treatment of IL-22 and S1P agonist further augmented the migration of MDA-MB231 cells (by up to 6.3-fold) and treatment with a S1P antagonist significantly interrupted this effect (Figure 3C,D). These findings emphasized the distinct role of IL-22 in breast cancer cell migration. 

Matrix metalloproteinase-2 (MMP-2) and MMP-9 have been suggested to promote the invasion of breast cancer cells [30,31] and we thus reasoned that these factors could be mediators of the invasive potential of breast cancer cells through IL-22. Although we observed that IL-22 did not induce MMP-2 enzymatic activity (data not shown), it did augment the secretion of MMP-9 protein from MDA-MB231 cells, an effect that was enhanced by a S1P agonist (Figure 3E), and effectively suppressed by a S1P antagonist (Figure 3E).

The IL-22 stimulation of MSCs increased the invasive potential of MDA-MB231 cells which was suppressed by the S1P antagonist (Figure 3F), again indicating that the IL-22 cytokine has a promoting effect on breast cancer cell migration and invasion via S1P signaling. Moreover, exposure of the cells to an MMP-9 inhibitor significantly blocked the IL-22-induced invasive capacity of MDA-MB231 cells towards MSCs (Figure 3G). Collectively, these results suggest that IL-22 enhances the invasive capacity of breast cancer cells via the induction of MMP9 expression, as well as via S1P signaling.

### 3.4. IL-22-Induced MCP-1 in MSCs Promotes Macrophage Chemotaxis

**Figure 4:**
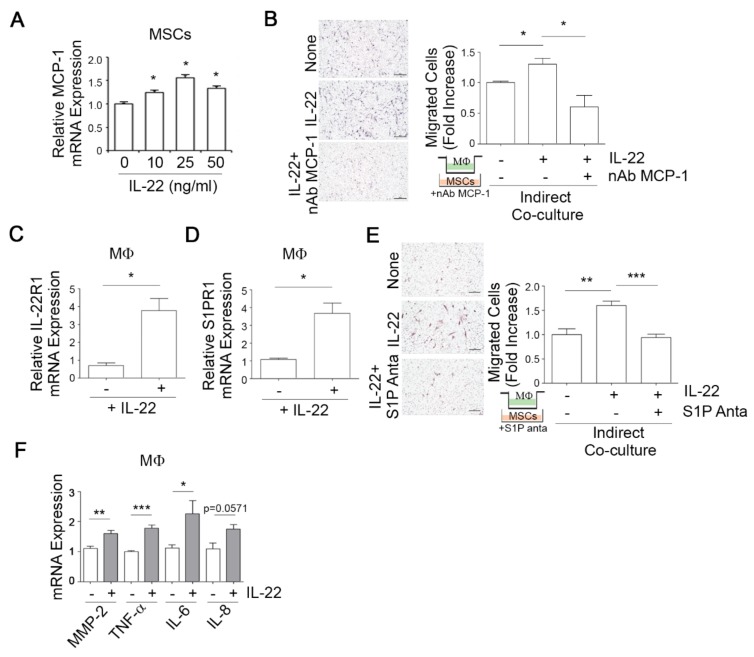
IL-22-induced monocyte chemoattractant protein 1 (MCP-1) in MSCs increases macrophage infiltration. (**A**) IL-22 induces the expression of MCP-1 in human bone marrow-derived MSCs. MSCs were stimulated with IL-22 for 24 h and then MCP-1 mRNA expression was analyzed by qPCR. (**B**) Macrophage infiltration is dependent on IL-22-induced MCP-1 in MSCs. MSCs were stimulated with IL-22 for 24 h in the lower Transwell chamber for preparation of the conditioned medium from IL-22-treated MSC cultures. Bone marrow-derived macrophages (BMMs) were loaded onto the upper chamber and the Transwell devices were inserted into the cultured lower wells of the plates with or without neutralizing antibody against MCP-1 (nAb MCP-1, 2 μg/mL). Migration was then assessed by hematoxylin staining. Mφ, macrophages. (**C**,**D**) Macrophages were treated with IL-22 and IL-22R1 (**C**) and S1PR1 (**D**) mRNA expression was then analyzed by qPCR. (**E**) Macrophages were loaded into the upper chamber of a Transwell system and MSCs were added to the lower chamber. For inhibition experiments, these cells were co-cultured with or without 1 μM of S1PR antagonist. After 24 h, the number of cells that had migrated through the matrigel matrix was analyzed (left) and quantified (right). Representative invasive images from the co-culture system are shown. (**F**) Macrophages were treated with IL-22 for 24 h and then MMP-2, TNF-α, IL-6, and IL-8 mRNA expressions were analyzed by qPCR. * *p* < 0.05, ** *p* < 0.005, *** *p* < 0.001 vs. vehicle control. Data are presented as the mean ± standard deviation from three independent experiments. Comparisons were performed using ANOVA.

Macrophage recruitment is known to be an important determinant of tumor metastasis [32]. We therefore assessed whether IL-22 could increase the chemotactic migration of macrophages toward MSCs. Given the well-known effect of MCP-1 in macrophage chemotaxis, human bone marrow-derived MSCs were stimulated with IL-22 for 24 h, resulting in increased MCP-1 mRNA expression as determined by qPCR (Figure 4A). In a subsequent Transwell assay, MSCs stimulated with IL-22 were incubated in the lower chamber and macrophages were loaded into the upper chamber with or without a neutralizing antibody against MCP-1 (nAb MCP-1). IL-22 was observed to promote the chemotactic migration of macrophages to MSCs and neutralizing antibody against MCP-1 completely abolished this effect (Figure 4B), suggesting that IL-22 induces the migration of macrophages to MSCs by upregulating MCP-1 expression in MSCs. 

We examined whether the IL-22 dependent induction of S1PR1 expression in MDA-MB231 breast cancer cells also occurs in macrophages. Indeed, we noted in macrophages that IL-22 exposure led to increased IL-22R1 transcript levels (Figure 4C), accompanied by an elevation in S1PR1 mRNA (Figure 4D), as revealed by qPCR. IL-22 treatment also induced the migratory potential of macrophages towards MSCs, an effect which was completely blocked by treatment with the S1P antagonist (Figure 4E). In addition, IL-22 treatment increased the expression of MMP-2, TNF-α, and IL-6 in macrophage (Figure 4F). These results suggest that IL-22 has a promoting effect on macrophage migration through S1P signaling.

### 3.5. Prognostic Value of the IL-22R1, S1PR1, and MMP-9 Expression Levels in Breast Tumors

**Figure 5:**
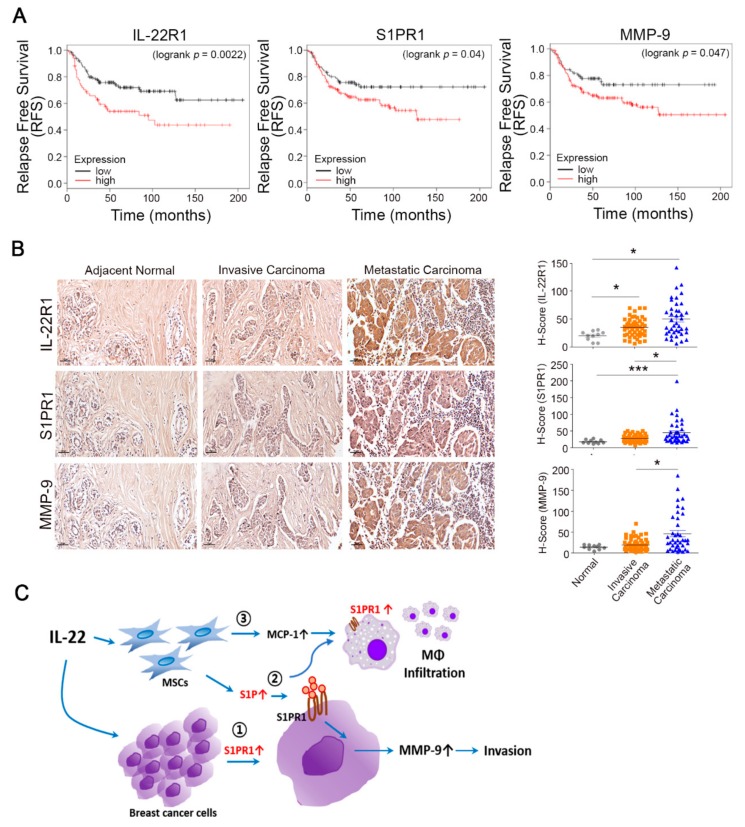
Prognostic value of the IL-22R1, S1PR1, and MMP-9 expression levels in breast tumors. (**A**) Relapse free survival curve of tumor IL-22R1, S1PR1, and MMP-9 expression in breast cancer patients (*n* = 425). The breast tumor IL-22R1, S1PR1, and MMP-9 levels were negatively associated with the overall survival or relapse free survival of breast cancer patients, as demonstrated by the Kaplan–Meier survival curves. The significance of these associations was determined using the log rank test. (**B**) The expression of IL-22R1, S1PR1, and MMP-9 protein was determined by immunohistochemical analysis (IHC) in normal, invasive breast carcinoma, and metastatic breast carcinoma specimens. Representative IHC photomicrographs are shown. Scale bar, 500 μm. H-scores determined by multiplying the staining intensity by the percentage of positive tumor cells. The expression patterns in the tested samples (100 breast tumor specimens) are shown. * *p* < 0.05, *** *p* < 0.001 vs. indicated group. (**C**) Schematic diagram of the proposed mechanism by which IL-22 regulates breast cancer progression.

Given the elevated expression of IL-22R1 and S1PR1 in aggressive breast cancer, we analyzed the associations between the IL-22R1 and S1PR1 signatures and relapse free survival time in a cohort of 425 human breast cancer patients. These subjects were divided into two subgroups based on the transcript levels of the genes. Kaplan–Meier survival analyses revealed that the high expression of IL-22R1 (*p* = 0.0022) and S1PR1 (*p* = 0.04) is associated with poor survival (Figure 5A). The high levels of the two genes were also associated with a concomitant lower rate of relapse free survival via elevated MMP-9 expression in human breast cancer patients (*p* = 0.047, Figure 5A). In comparison, the overall survival curves indicated that the IL-22R1 signature is not associated with poor survival outcomes in breast cancer patients, although cases with an elevated expression of S1PR1 exhibited a lower overall survival rate (*p* = 0.016, Figure S4). Our findings thus indicated that elevated IL-22R1, S1PR1, and MMP-9 expression may result in poorer survival in cases of relapsed breast cancer. 

Next, the role of IL-22R1, S1PR1, and MMP-9 in breast cancer progression and the potential prognostic value of assaying these factors were evaluated through the immunohistological staining of adjacent normal, invasive breast carcinoma, and metastatic breast carcinoma tissues. IL-22R1, S1PR1, and MMP-9 were found to be predominantly expressed in metastatic breast carcinoma specimens but only weakly detected in normal tissues and invasive breast carcinoma (Figure 5B, left). Notably, metastatic breast carcinomas expressed elevated S1PR1 compared to invasive metastatic breast carcinomas, indicating that elevated S1PR1 may correlate with metastatic breast tumors but not in primary breast tumors (Figure 5B, right). These results suggest that elevated IL-22R1, S1PR1, and MMP-9 may be associated with a more advanced tumor and thus be related to a poorer prognosis in breast cancer patients.

## 4. Discussion

Current therapeutic strategies for breast cancer, including neoadjuvant chemotherapy, have shown limited efficacy in cases of metastatic breast cancer experiencing disease recurrence [33]. Hence, a clear understanding of the underlying mechanism of interaction between metastatic breast cancers and their niche is of substantial interest with regard to therapeutic advances. We show from our current analyses that the IL-22-IL-22R1-linked axis is a critical component of the invasive phenotype in aggressive breast cancer. Indeed, both IL22R1 and S1PR1 expression is elevated in invasive breast tumor specimens and bone metastatic breast cancer cells. Of particular note also, IL-22R1 expression was found to positively correlate with the S1PR1 level at mineral sites including bone (Figure 1I, *p* < 0.0001 in bone and *p* < 0.001 in brain). Our finding that the inhibition of S1P1R signaling with an S1P antagonist suppressed the IL-22-evoked increase in breast cancer cell invasiveness by reducing MMP-9 activity (Figure 3) suggests that enhancement of the IL-22R1-S1PR1-linked axis via IL-22 stimulation may be involved in the increased metastatic potential of advanced breast cancer to bone niches (Figure 5C).

Our present results are consistent with the findings of previous reports demonstrating that S1P is involved in cancer progression by contributing to cell proliferation, migration, invasion, angiogenesis, and metastasis [34,35,36]. Indeed, the plasma S1P level was shown to be augmented in ovarian cancer patients [37] and to be associated with an increased risk of developing lung cancer [38]. In addition, an elevated level of S1P was observed in cancer interstitial fluid compared with surrounding normal tissue [22]. This suggested that this protein could affect the tumor microenvironment not only as an initial metastasis signal but also as a supplier of additional factors that promote metastasis. Nonetheless, we noted from our current experiments that IL-22 induced the expression of sphingosine kinase and S1P production in MSCs, which are interstitial stromal cells, but not in MDA-MB 231 cells. In addition, we observed that IL-22 promoted the invasive activity of breast cancer cells by inducing MMP-9 to facilitate the accumulation of macrophages (Figure 3 and Figure 4), thereby providing an initial signal for cancer metastasis. Hence, the IL-22 cytokine may be a critical mediator of the osteolytic relationship between the bone microenvironment and metastatic breast cancer.

IL-22 has recently been proposed as a target for cancer therapy [9] as its increased expression has been reported in colonic [39], gastric [20], and hepatocellular cancer [19], as well as in small- and large-cell lung carcinoma [40]. Excessive IL-22 levels were also noted in a prior study in infiltrated leukocytes of a hepatocellular carcinoma patient [19]. In our current study, we found that IL-22 was produced from T lymphocytes under pathological inflammatory conditions in the LPS-induced bone loss mouse model (data not shown). In accordance with this finding, IL-22 was found previously to be increased in synovial tissues and serum, which correlated with the inflammatory disease severity, including arthritis rheumatism [41,42]. Increased IL-22 further promotes the proliferation of synovial fibroblasts and induces the production of MCP-1, which attracts monocytes to the synovium [43]. Notably, our present results show that IL-22 signaling pathway significantly enhanced the invasive potential of breast cancer cells and increased MCP-1 production from MSCs, inducing macrophage accumulation (Figure 3 and Figure 4). Brizuela et al. have also reported that S1P acts as a paracrine growth factor that is released from osteoblasts under inflammatory conditions [44]. Furthermore, we found that the IL-22R1 and S1PR1 mRNA levels are profoundly elevated and significantly associated with breast cancer tissues that have developed bone/brain metastasis. We further revealed that patients with IL-22R^high^ breast cancers displayed poorer survival rates compared to those with IL-22R^low^ tumors (Figure 5) and that the IL-22R level exhibited a positive relationship with S1PR1 expression (Figure 1). The evidence to date thus indicates that IL-22 can be produced in inflammatory states and stimulate the S1P pathway, thereby accelerating the development of breast cancer metastases. However, the specific mechanism by which IL-22R1-S1PR1-linked signals regulate the bone distant metastasis of aggressive breast cancer cells in vivo remains to be further investigated. 

In conclusion, we have here elucidated a mediatory role of an IL-22-linked signal axis in metastatic breast cancer cells and uncovered mechanisms underlying the potential crosstalk of cancer cells with host cells within the local tumor environment (Figure 5C). We showed that IL-22 enhances S1PR1 expression in invasive metastatic breast cancer cells (①) and induces S1P expression and release in MSCs (②) to promote the migratory and invasive potential of breast cancer cells towards MSCs. In addition, IL-22 induces MCP-1 in MSCs and S1PR1 in macrophages and thereby promotes macrophage infiltration (③) (Figure 5C). This axis thereby contributes to the osteolytic characteristics of metastasized breast cancer. The pathological functions of this IL-22-linked axis may be a promising therapeutic target for breast cancer bone metastases.

## Supplementary Materials

The following are available online at https://www.mdpi.com/2073-4409/9/1/131/s1, Figure S1: Comparison of CD68, MCP-1, CD14, IL-22, and S1PR2-5 expression in breast tumors. (A, B) CD68, MCP-1, and CD14 mRNA expression levels in the luminal and basal-like/triple-negative subtypes of human breast cancers from the GSE12777 (A) and GSE65194 (B) dataset were compared using the chi-square test. (C, D) IL-22, S1PR2-5 (C), CD68, MCP-1, and CD14 (D) mRNA expression levels in lung, brain, bone, and liver metastasis-positive human breast cancer mined from the GSE14020 dataset were compared. Values are expressed as a mean  ±  SD. Comparisons were performed with ANOVA (multiple groups). * *p* < 0.05, ** *p* < 0.005, *** *p* < 0.001 vs. corresponding controls. n.s., not significant. Figure S2: (A, B) MCF-7 (A) and HCC1954 (B) cells were treated with IL-22 for 24 h and IL-22R1 and S1PR1-4 transcript levels were analyzed by qPCR. **p* < 0.05, ** *p* < 0.005, *** *p* < 0.001 vs. corresponding controls. Figure S3: (A) pERK, ERK, pSTAT3, STAT3, pAKT, and AKT protein expressions were determined by immunoblotting assay in low metastatic (MCF-7, T47D, MDA-MB453, and SKBR3) and highly metastatic (HCC1954, MDA-MB231, and MDA-MB231MT) breast cancer cell lines. (B) MDA-MB231 cells were transfected with control siRNA or siRNA targeting IL-22R1 and treated with IL-22 for the indicated times. pSTAT3, STAT3, p-p38, p38, pERK, ERK, pJNK, JNK, pAKT, and AKT protein expressions were determined by immunoblotting assay. (C) MCF-7 and MDA-MB231 cells were treated with IL-22 in the presence or absence of DMSO, PD98059 (ERK inhibitor), STAT3i (STAT3 inhibitor), LY2940002 (PI3K inhibitor), SB203580 (p38 inhibitor), or SP600125 (JNK inhibitor) for 24 h and S1PR1 transcript level was analyzed by qPCR. * *p* < 0.05, ** *p* < 0.005 vs. corresponding control. Figure S4: Overall survival curve for tumor IL-22R1, S1PR1, and MMP-9 expression in breast cancer patients. (*n* = 425). The significance of the associations was determined by log rank test. Table S1: Primers used in this study.

## Abbreviations

BMMs	Bone marrow-derived macrophages
CM	conditioned media
FBS	fetal bovine serum
GAPDH	glyceraldehyde-3-phosphate dehydrogenase
HCC	hepatocellular carcinoma
IL-22	interleukin 22
IL-22R1	interleukin-22 receptor 1
MMP-2	matrix metalloproteinase-2
MMP-9	matrix metalloproteinase-9
MSCs	mesenchymal stem cells
MCP-1	monocyte chemoattractant protein 1
PBS	phosphate-buffered saline
S1P	sphingosine-1-phosphate
S1PR1	sphingosine-1-phosphate receptor 1
