# Supplementary material for: Interleukin-22 Mediates the Chemotactic Migration of Breast Cancer Cells and Macrophage Infiltration of the Bone Microenvironment by Potentiating S1P/SIPR Signaling
Source: Cells. 2020 Jan 6;9(1):131. doi: 10.3390/cells9010131 (PMC7017200; doi:10.3390/cells9010131)
Supplement: Supplementary file 1 [file cells-09-00131-s001.pdf]

Supplementary Information

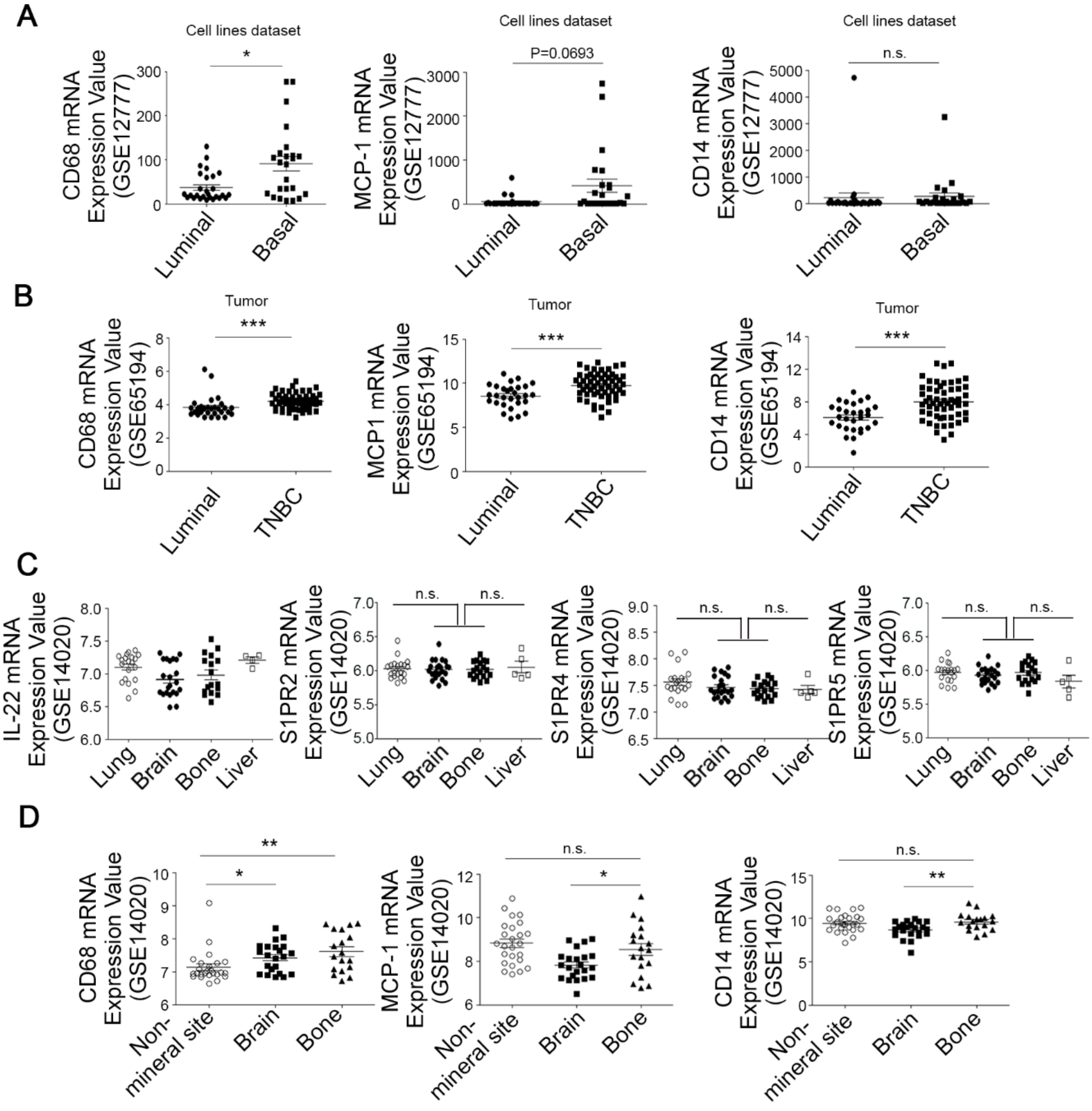

Figure S1

Figure S1. Comparison of CD68, MCP-1, CD14, IL-22, and S1PR2-5 expression in breast tumors. (**A**, **B**) CD68, MCP-1, and CD14 mRNA expression levels in the luminal and basal-like/triple-negative subtypes of human breast cancers from the GSE12777 (**A**) and GSE65194 (**B**) dataset were compared using the chi-square test. (**C, D**) IL-22, S1PR2-5 (**C**), CD68, MCP-1, and CD14 (**D**) mRNA expression levels in lung, brain, bone, and liver metastasis-positive human breast cancer mined from the GSE14020 dataset were compared. Values are expressed as a mean ± SD. Comparisons were

performed with ANOVA (multiple groups). *P < 0.05, **P < 0.005, ***P < 0.001 vs corresponding controls. n.s., not significant.

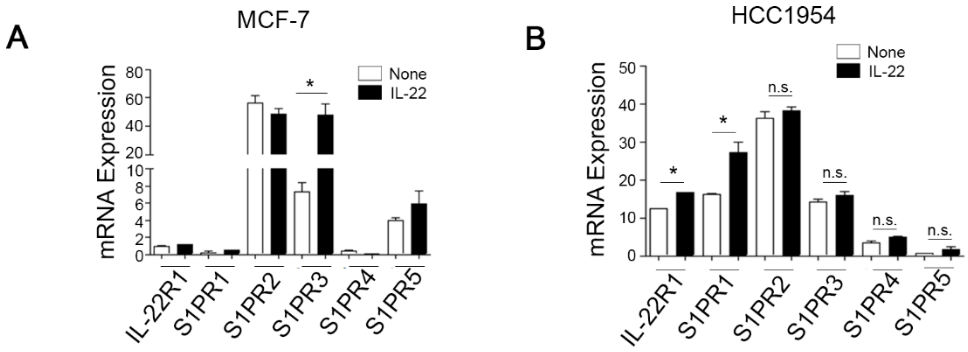

Figure S2

Figure S2. (**A**, **B**) MCF-7 (**A**) and HCC1954 (**B**) cells were treated with IL-22 for 24 h and IL-22R1 and S1PR1-4 transcript levels were analyzed by qPCR. *P < 0.05 vs corresponding controls.

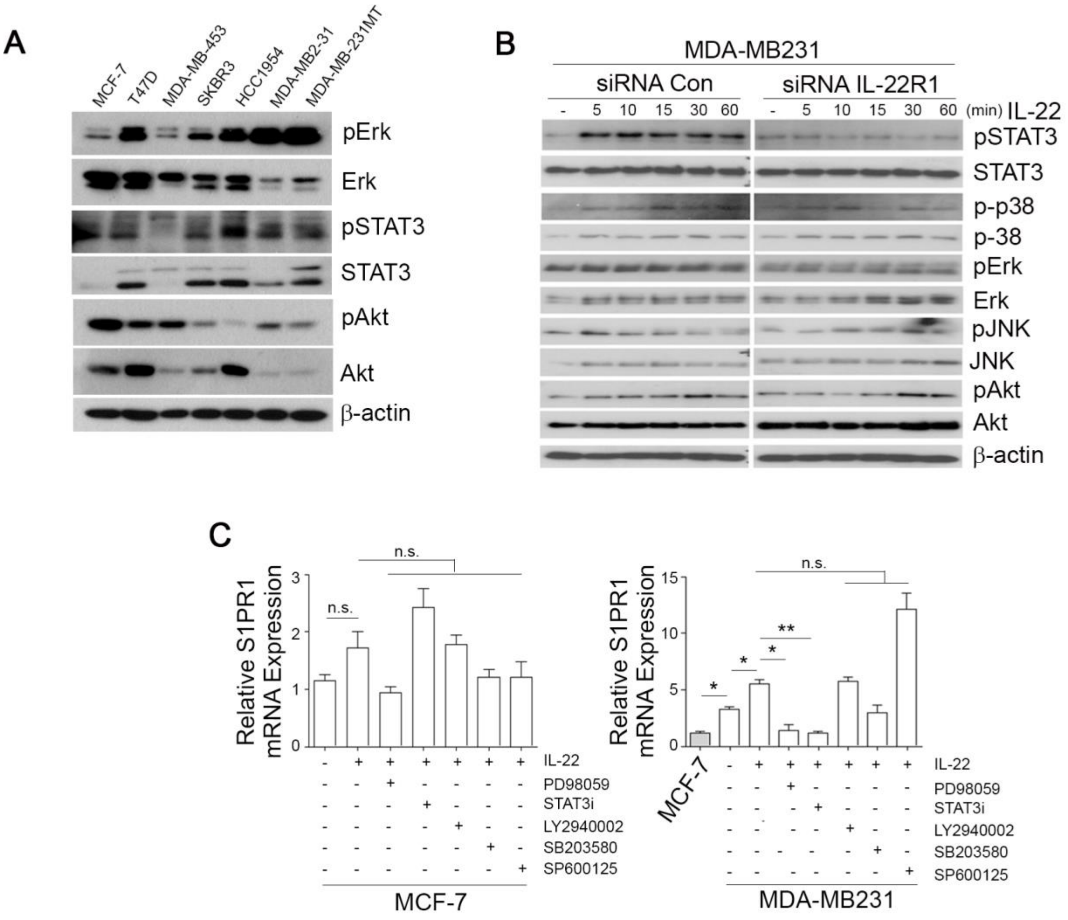

Figure S3

Figure S3. (**A**) pERK, ERK, pSTAT3, STAT3, pAKT, and AKT protein expressions were determined by immunoblotting assay in low metastatic (MCF-7, T47D, MDA-MB453, and SKBR3) and highly metastatic (HCC1954, MDA-MB231, and MDA-MB231MT) breast cancer cell lines. (**B**) MDA-MB231 cells were transfected with control siRNA or siRNA targeting IL-22R1 and treated with IL-22 for the indicated times. pSTAT3, STAT3, p-p38, p38, pERK, ERK, pJNK, JNK, pAKT, and AKT protein expressions were determined by immunoblotting assay. (**C**) MCF-7 and MDA-MB231 cells were treated with IL-22 in the presence or absence of DMSO, PD98059 (ERK inhibitor), STAT3i (STAT3 inhibitor), LY2940002 (PI3K inhibitor), SB203580 (p38 inhibitor), or SP600125 (JNK inhibitor) for 24 h and S1PR1 transcript level was analyzed by qPCR. *P < 0.05, **P < 0.005 vs corresponding control.

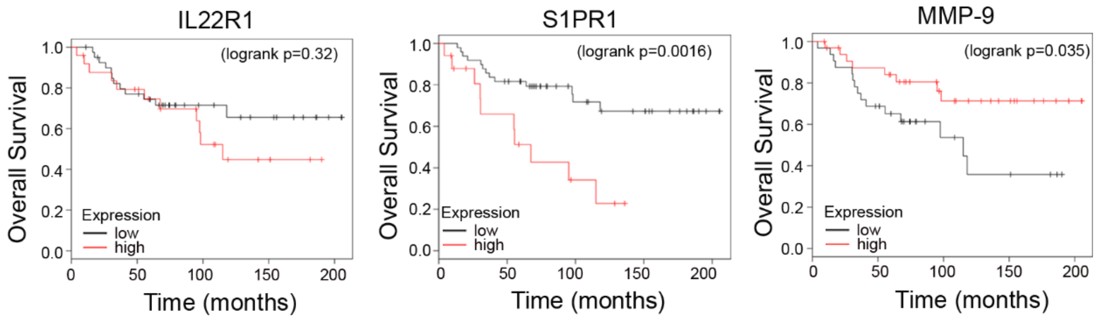

Figure S4

Figure S4. Overall survival curve for tumor IL-22R1, S1PR1, and MMP-9 expression in breast cancer patients. (n=425). The significance of the associations was determined by log rank test.

Table S1. Primers used in this study

Gene name	Primer sequences	
human IL-22R1	Forward	5'-GACTCCATTTGGGTCATCGC-3'
	Reverse	5'-CCTGTCACCGTGTGTCATCA-3'
human S1P1	Forward	5'-TCCATGTAAACTGGGTCAAG-3'
	Reverse	5'-AAAGGTGCTGTAGGGGTTAG-3'
human S1P2	Forward	5'-TTTTAAAATTGGGACAGGGT-3'
	Reverse	5'-TTCTCCACAGGATTTAGCAA-3'
human S1P3	Forward	5'-ATGGCATTTGCTCTTGTTTA-3'
	Reverse	5'-TATTTTTCCCTTAACCCAGC-3'
human S1P4	Forward	5'-AACTGTGGGTATGACTCTGG-3'
	Reverse	5'-ATACAGTTGGAACAGTTGGG-3'
human S1P5	Forward	5'-ACCATTCCTAGATCGAACCGT-3'
	Reverse	5'-CACCACCCCGAAGATGAACAT-3'
human GAPDH	Forward	5′-TGTTGCCATCAATGACCCCTT-3′
	Reverse	5′-CTCCACGACGTACTCAGCG-3′
